# A vertebrate model to reveal neural substrates underlying the transitions between conscious and unconscious states

**DOI:** 10.1038/s41598-020-72669-1

**Published:** 2020-09-25

**Authors:** Victoria M. Bedell, Qing C. Meng, Michael A. Pack, Roderic G. Eckenhoff

**Affiliations:** 1 Department of Anesthesiology and Critical Care, University of Pennsylvania, Philadelphia, USA; 2 Department of Medicine, University of Pennsylvania, Philadelphia, USA

**Keywords:** Neurochemistry, Behavioural genetics, Sleep

## Abstract

The field of neuropharmacology has not yet achieved a full understanding of how the brain transitions between states of consciousness and drug-induced unconsciousness, or anesthesia. Many small molecules are used to alter human consciousness, but the repertoire of underlying molecular targets, and thereby the genes, are incompletely understood. Here we describe a robust larval zebrafish model of anesthetic action, from sedation to general anesthesia. We use loss of movement under three different conditions, spontaneous movement, electrical stimulation or a tap, as a surrogate for sedation and general anesthesia, respectively. Using these behavioral patterns, we find that larval zebrafish respond to inhalational and IV anesthetics at concentrations similar to mammals. Additionally, known sedative drugs cause loss of spontaneous larval movement but not to the tap response. This robust, highly tractable vertebrate model can be used in the detection of genes and neural substrates involved in the transition from consciousness to unconsciousness.

## Introduction

Every year over 200 million major surgeries involving anesthesia are performed worldwide^1^. Anesthesia is a spectrum of states, ranging from light “conscious” sedation to fully unresponsive drug-induced coma. Different doses of the same drugs, or different drugs can be used to achieve these states. These drugs are clinically classified as inhaled or injectable, and then by what are thought to be the principle molecular targets (e.g., glutamatergic or GABAergic), although many targets likely contribute^2^. Targets include a wide range of voltage and ligand gated ion channels, transporters, second messenger and mitochondrial proteins^3,4^, with the injectable drugs being somewhat more selective^5–8^.

Our mechanistic knowledge has been derived from many different experimental models, including biochemical, cell culture, and non-human animal models (worms^9^, flies^10^, tadpoles^11^, mice^12^, rats and dogs^13^). While invertebrates have easily manipulated genomes and simplified neural structures, they do not have the complex neural structures of vertebrates. Rats, dogs or mice have been the primary vertebrate model, but they are expensive, and their progeny are limited. The zebrafish is vertebrate model organism that combines the genomic manipulability of the invertebrates with the neural structures of a vertebrate. Further, the zebrafish larvae transparency and small size allow for imaging of neural structures that range from the whole brain to a single cell within a live organism. The zebrafish is a genetically diverse organism and cannot be bread to isogenecity. Therefore, the large clutch size provides an adequate number of animals to replicate of the data^14^. Thus, the zebrafish is an ideal model to study neuropharmacology^15^.

Defining the response to anesthetics in animal models, and in humans^16^, has traditionally been reliant upon movement, either spontaneous or elicited^9,17^. Examples of elicited movement include a startle response to a tap, poke, pinch or a change in position (e.g., a righting reflex^18–21^). For all the organisms used, the outcome is binary; either the animal moves or does not^20^. Continuous measures of anesthesia are rare, usually relying on electrophysiology or imaging.

Here we report a simple, reproducible, continuous, quantitative model of anesthesia that includes light sedation to general anesthesia using movement endpoints in 5 days post-fertilization (dpf) larval zebrafish. Loss of spontaneous movement (SPONT) models light sedation, movement in response to electrical stimulation (ELEC) models deep sedation and movement in response to a tap stimulus (TAP) models general anesthesia^22^.

## Results

### Establishing the zebrafish behaviors in 5 dpf larvae

**Figure 1:**
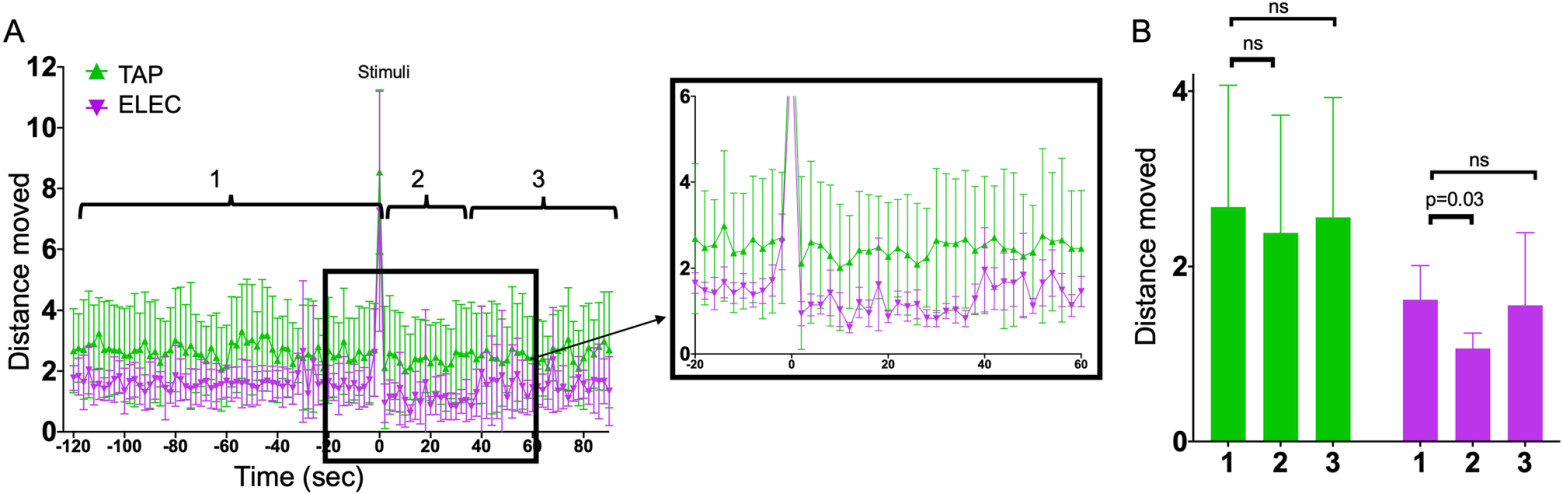
Zebrafish movement before and after TAP and ELEC. (**A**) The zebrafish were behaviorally equilibrated within the chambers used to induce stimuli. Prior to a stimulus (1) the distance moved over 2 s was stable in both chambers, which the circular tap wells having a higher average distance moved when compared to the rectangular ELEC chamber. At the 0 time point, the zebrafish larvae were stimulated with either tap or electrical shock. The following 36–40 s (block box magnification) the zebrafish with the TAP (green) moved normally whereas the ELEC demonstrated a decrease in movement (purple). (**B**) When averaging the 2 s of distance moved for the 120 s prior to the stimulus (1), the 40 s immediately following the stimulus (2) and 40–90 s after the stimulus, there is no significant difference in movement for the TAP (green). However, for the ELEC (purple), there is a statistically significant decrease in movement following the stimulus but the larvae recover back to baseline movement after approximately 40 s. Five replicates where assessed with 8–12 larvae per replicate.

**Figure 2:**
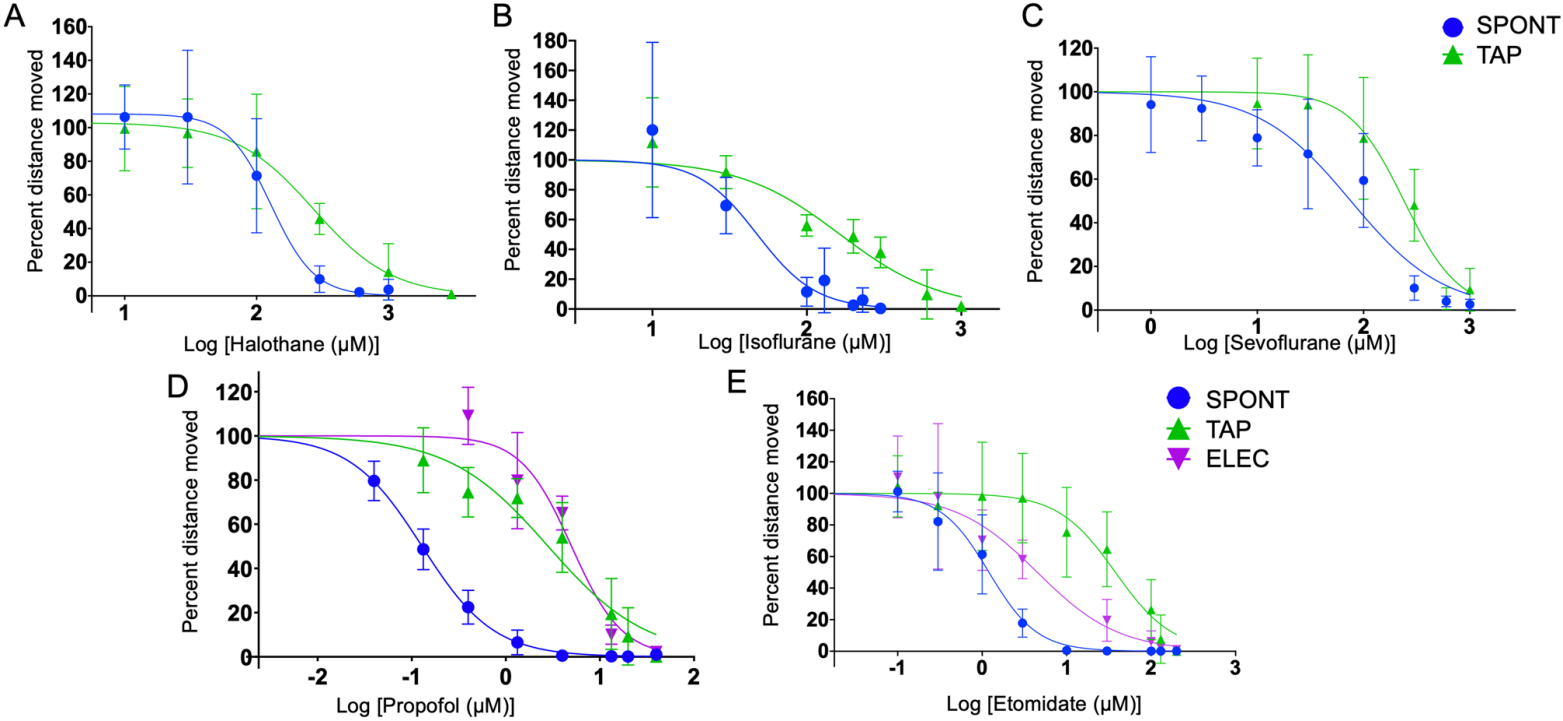
Hill plots of the inhaled and IV anesthetics. Zebrafish respond to inhaled anesthetics, halothane (**A**), isoflurane (**B**) and sevoflurane (**C**), and IV anesthetics, propofol (**D**) and etomidate (**E**), at physiological concentrations. For all anesthetics, the SPONT (blue) is lost at a lower concentration as compared to the TAP (green). For the IV anesthetics, ELEC (purple) is also lower than SPONT. Interestingly, although propofol TAP (green) and ELEC (purple), are similar, etomidate requires a sevenfold higher dose of drug for TAP versus ELEC. All graphs were created with 3–8 replicates per point and 15–18 zebrafish larvae used per replicate.

We used three different behavioral assays to study anesthesia in the zebrafish. First, we used loss of spontaneous movement (SPONT) as a surrogate sedation phenotype, as has been reported previously^23,24^. Second, we used loss of the startle reflex as a general anesthetic phenotype. There are two ways of triggering the startle reflex, through a light stimulus or a TAP stimulus. The light stimulus has been used previously^24^ and both sedative medications as well as general anesthetics caused loss of the movement response. Therefore, we decided to use the tap stimulus to be able to better differentiate between sedation and general anesthesia. Finally, we wanted to use loss of response to a noxious stimulus. Previous work demonstrated that some chemicals, such as mustard oil, were noxious to the larval zebrafish through activation of the TRPA1 chemosensor^25^. However, when we placed the larvae into three noxious stimuli, mustard oil (S Fig. 1a,b), low pH and high pH (S Fig. 2), we found a decrease in movement. Therefore, we decided to attempt to use the initial burst of movement when the larvae were exposed to a noxious stimulus. To do this, we decided to pursue a low-amplitude electrical stimulus (ELEC).

### Defining the behavior to the ELEC stimulus

Using our fabricated ELEC chamber, we compared movement of the zebrafish larvae both before and following the stimulus, to the movement of fish within the round TAP standard plates. The amount of SPONT with the ELEC plate was less than that seen within the TAP circular chambers (Fig. 1a). We were able to see a spike in movement after stimulus, with no difference in distance moved between the TAP and ELEC. This increase in movement was used in the subsequent anesthetic experiments.

Interestingly, following the ELEC there was a statistically significant decrease in movement (Fig. 1a,b) for 36–38 s that is very similar to the lack of movement seen when mustard oil was applied (S Fig. 1). This was followed by a return to normal movement (Fig. 1a,b). On the other hand, after the TAP stimulus, there was no decrease in movement (Fig. 1b).

### Zebrafish larvae responded to inhaled anesthetics at clinically relevant concentrations

**Figure 3:**
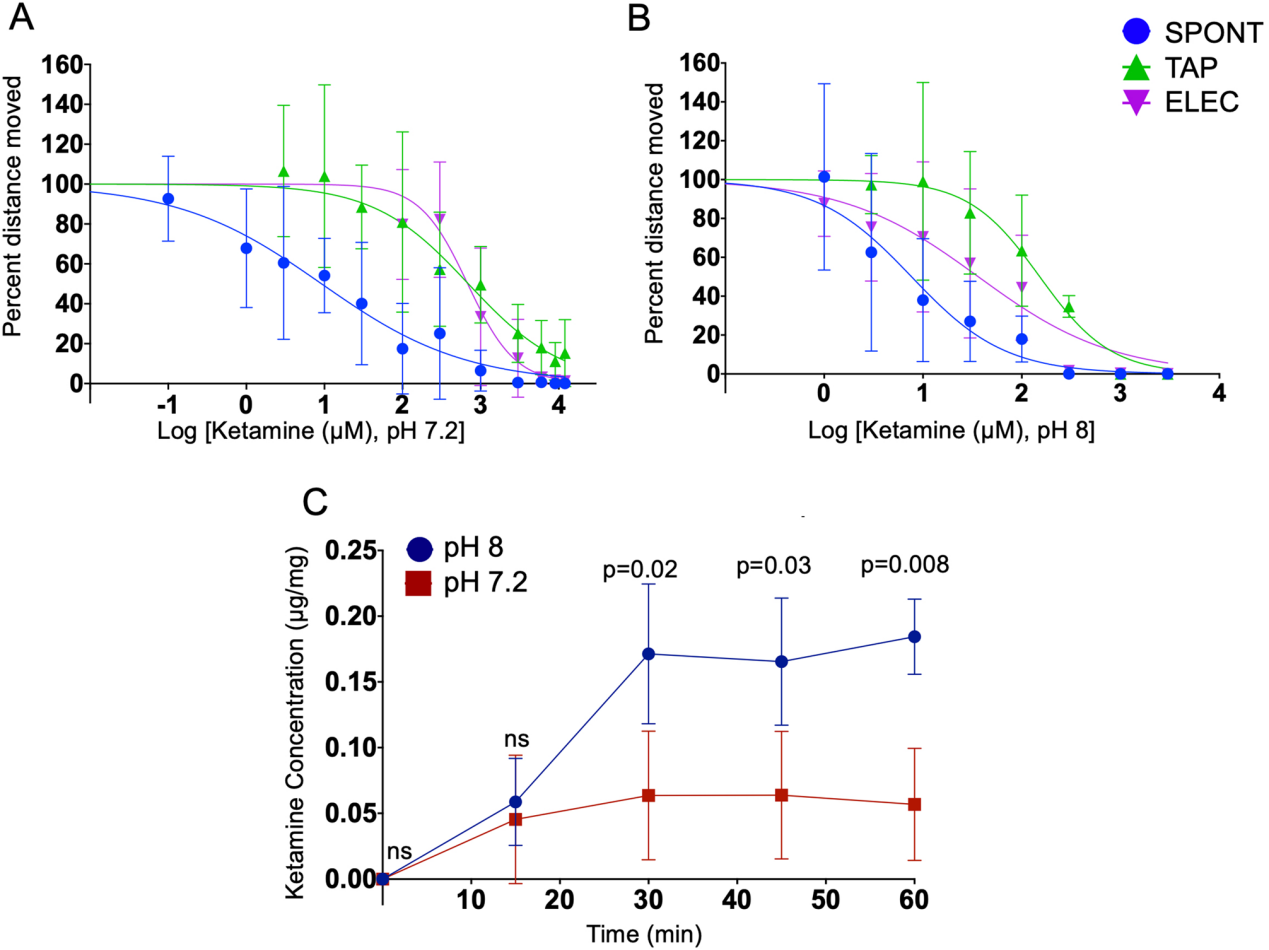
Ketamine hill plots and absorption. (**A**, **B**) Change in behavior for SPONT (blue), TAP (green) and ELEC (purple) required very high doses of ketamine at pH 7.2 (**A**) versus pH 8 (**B**). For the TAP (**A**, green), complete loss of movement was unable to be attained. Graph was created with a minimum of three replicates per point and 8–12 zebrafish larvae used per replicate. Ketamine at pH 8 (**B**) requires a fourfold higher concentration for TAP versus ELCT. (**C**) We used HPLC to determine the ketamine concentration in the zebrafish larvae over time. We found a significantly higher concentration of ketamine at pH 8 versus pH 7.2. Additionally, this shows that the ketamine equilibrated at approximately 30 min. Each point was replicated five times and contained 20–25 larval zebrafish per replicate.

After defining our behaviors, example movement data seen in S Fig. 3 a,b, we determined the sensitivities of 5 dpf larvae to general anesthetics. We began with the inhaled anesthetics, but only to the SPONT and TAP paradigms, since the ELEC apparatus could not contain the volatile drugs. We fit the data to standard Hill curves to calculate the median effective concentration as which 50% of the animals responded to the stimulus (EC50) for halothane, isoflurane and sevoflurane (Fig. 2a–c). We demonstrated that the EC50 for SPONT and TAP for all three inhaled anesthetics, were very similar to that of mammals, including humans. As expected, the SPONT EC50 was lower than that for TAP (Fig. 2a–c). The EC50 for each drug and both behaviors are summarized in Table 1. Interestingly, the range of movement response was very large at low concentrations of isoflurane. Some animals demonstrated increased movement, while others showed no change or decreased movement. The TAP, in particular, appeared to elicit a hyperactive movement phenotype (Fig. 2b). This enhanced variability was not seen at low concentrations of the other inhaled anesthetics.

**Table 1:** Summary of the EC50 for all drugs.

Drug	Behavior	EC50 (µM)	95% CI (µM)	pKa	7 dpf ZF (µM)^23^	Human (µM)
**Inhaled general anesthetics**						
Halothane	SPONT	132.2	101.9–188.2	N/A		
	TAP	329.6	208.7–497.4			
	Prior studies					250^26^
Isoflurane	SPONT	41.6	48.4–63.9	N/A		
	TAP	180.1	146.2–220.8			
	Prior studies					320^26^
Sevoflurane	SPONT	75.9	49.6–113.8	N/A		
	TAP	240.5	168.7–328.5			
	Prior studies					522^27^
**IV general anesthetics**						
Propofol	SPONT	0.097	0.09–0.11	11	Spont: 0.12	
	TAP	2.75	1.7–4.3			
	ELEC	3.78	2.8–5.0			
	Prior studies				Light: 0.7	0.4–2^26–28^
Etomidate	SPONT	1.2	0.92–1.6	4.2	Spont: 0.03	
	TAP	28.2	18.2–43.3			
	ELEC	3.95	2.0–8.5			
	Prior studies				Light: 0.6	1.2–10^29^
Ketamine, pH 8	SPONT	6.52	2.5–19	7.5	Spont: 8	
	TAP	156.3	72.4–313.6			
	ELEC	36.7	19.7–66.8			
	Prior studies				Light: 50	10^30^
**Sedative**						
Dexmedetomidine	SPONT	0.0087	0.007–0.011	7.1	Spont: 0.01	
	TAP	N/A	N/A			
	ELEC	4.69	2.3–9.5			
	Prior studies				Light: 0.4	

The EC50 for all drugs tested and for each behavior was obtained with the 95% confidence intervals. The pKa for each drug is shown, demonstrating that only the ketamine had a pKa higher than the pH 7.2 of the E3. Data from Yang et al.^23^ in 7 dpf zebrafish larvae demonstrates similar concnetrations for SPONT with lower concentrations for the stimulus. Both our data and the 7 dpf data are within clinical range when compared to human data.

### Zebrafish larvae responded to intravenous anesthetics at clinically relevant concentrations

Next, we determined the zebrafish sensitivity to three intravenous anesthetics, propofol, etomidate and ketamine. Two of these drugs, propofol and ketamine, are used both as sedative agents and as general anesthetics. Each drug was studied using the three behaviors defined above, SPONT, TAP and ELEC, and the data are shown in Figs. 2d,e and 3, and the EC50 is summarized in Table 1.

While defining the ketamine dose response curves, we found that ketamine in normal E3 embryo water (pH of 7.2) the EC50 values were high for all three behaviors (S Table 1). Therefore, we hypothesized that increasing the E3 pH to above ketamine’s pKa of 7.5 would increase the concentration of non-ionized drug, allowing greater uptake and thereby reduce EC50. First, to rule out an independent effect of altered pH, we examined a wide range of pH values, and found no significant change in baseline spontaneous movement at a pH range of 5–8.5 (S Fig. 2). Therefore, we compared the EC50 of ketamine at pH 7.2 (Fig. 3a) to that at pH 8 (Fig. 3b), and found the EC50 at pH 8 to be significantly lower (S Table 1). To confirm that these behavior responses were due to differential uptake of drug, we measured total zebrafish ketamine content using HPLC in 15-min increments at both pHs. There was significantly less ketamine in the zebrafish after 30 min at pH 7.2 versus pH 8 as predicted (Fig. 3c). Unexpectedly, for both etomidate and ketamine, the response to TAP had a higher EC50 as compared to the ELEC, whereas in propofol they were similar.

### Differentiating between sedation and general anesthesia

**Figure 4:**
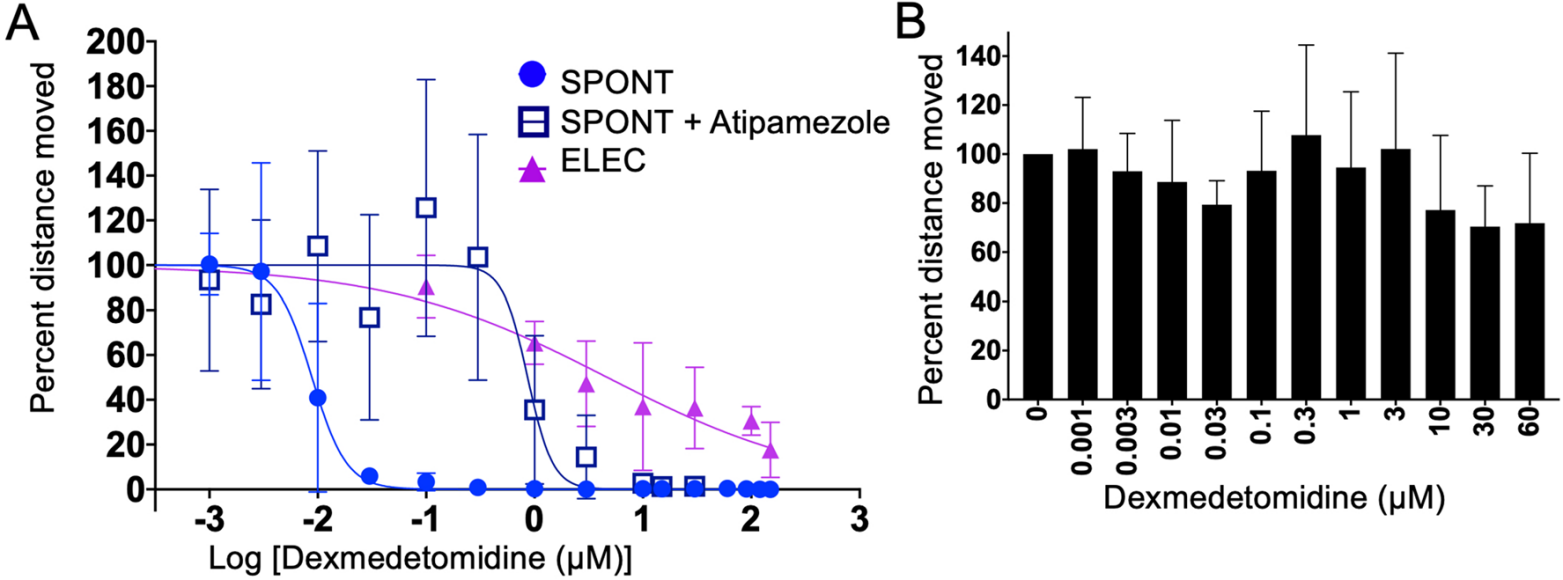
Hill plots of a sedative drug. Zebrafish respond to dexmedetomidine at physiological concentrations for loss of SPONT (**A**, light blue circle). This response is able to be reversed using an alpha-2 adrenergic antagonist, atipamezole (**A**, dark blue square) at 100 µM. There is a decrease in movement seen in the ELEC (**A**, purple triangle). However, even at the highest doses, we were unable to achieve complete loss of movement. (**B**) There is no significant difference seen with this drug using the TAP (p = 0.4). All graphs were created with 3–8 replicates per point and 8–12 zebrafish larvae used per replicate.

While using the general anesthetics listed above, we defined a sedation phenotype as a loss of SPONT with general anesthesia being loss of TAP or ELEC. For all the drugs, the SPONT EC50 was significantly lower than the other behaviors. As seen above, for etomidate and ketamine, the TAP had a higher EC50 as compared to the ELEC (Table 1). To better understand where the response to the ELEC falls along the sedation-anesthesia continuum, we tested dexmedetomidine, an alpha2-adrenergic receptor agonist. This drug is sedative but even at high dose does not produce general anesthesia. Additionally, it has some analgesic properties. Dexmedetomidine produced a loss of SPONT with an EC50 of 0.0087 µM (0.0068–0.011 µM 95% CI) (Fig. 4a, light blue; Table 1). The sensitivity of SPONT to dexmedetomidine was dramatically reduced by co-incubation with a known alpha2 adrenergic receptor inhibitor, atipamezole; with the EC50 increasing 100-fold to 0.86 µM (0.52–1.5 µM 95% CI) (Fig. 4a, dark blue). We saw no loss in movement to TAP up to 150 µM dexmedetomidine (Fig. 4b), but we found that there was an 80% decrease in movement to ELEC (Fig. 4a, purple) at 150 µM dexmedetomidine. The EC50 of the ELEC was 4.7 µM (2.3–9.5 µM 95% CI), which is much higher dosing than is used in humans. These results suggest that mitigation of the ELEC stimulus might be considered deep-sedation.

## Discussion

Here we show that a vertebrate model system, the larval zebrafish, can provide a simple, robust, quantitative model of drug-induced behaviors that should be easily reproduced in other laboratories. We examined motion in response to three stimuli; none (SPONT), TAP and ELEC, a paradigm of progressively more noxious stimulus^22,24^. Using our data, we can define a loss of SPONT as sedation, ELEC as deep sedation and TAP as general anesthesia. However, these definitions must remain tentative until more objective measures become available to define the continuum of sedation to anesthesia.

There are many key features of the model that we decided upon, including using the larval animal, standardizing the movement assays, deciding upon the noxious stimulus and exploring the effect of pH on a 5 dpf larva. First, the zebrafish is a larval, developmental model. We took advantage of the small size, rapid growth, large clutch size and transparency of the zebrafish larvae instead of adults. Five dpf larvae have a yolk sac to provide nutrition but also display complex behaviors. Drugs equilibrate into the fish via simple diffusion, bypassing many of the pharmacokinetic issues that complicate studies in larger animals. The 2 dpf zebrafish also has a robust startle response, but have less mature neural structures, and lower expression of the transporters that establish a mature chloride gradient^31^. Although the 6–7 dpf zebrafish brain has greater complexity, the yolk sac is becoming depleted, allowing nutritional status to complicate movement endpoints^32^. Additionally, the animal is larger, making imaging more difficult. Therefore, 5 dpf animal have the best balance of neural complexity and experimental tractability.

Second, we standardized the movement assays. Different wild type strains and ages of zebrafish move different distances in response to stimuli^33^. Additionally, there is high genetic variation in the same strain of fish^34,35^. To reduce these sources of variation, we normalized the movement to no drug control in the same clutch on the same day.

Third, we developed a noxious stimulus. We desired a noxious stimulus that could approximate a surgical stimulus, but avoid tissue damage. A touch or poke stimulus, is feasible but not readily reproducible or scalable for screens. Noxious stimuli previously used were chemicals or environmental changes, such as mustard oil, capsaicin, cold, heat and increased salinity^25,36,37^. Again, these are more difficult to scale, and often produce biphasic responses.

Electrical stimulation can be applied to the larvae in a defined, constant burst^38,39^ that can be varied to explore dose–response effects. ELEC allows a higher throughput with less operator bias than other noxious stimuli, and will allow simultaneous imaging and mapping of neural pathways, so that comparisons to the TAP reflex pathways^40^ can occur.

Finally, we explored the effect of pH on compound diffusion. Of the drugs studied here, only ketamine is charged at normal E3 pH (7.2), suggesting lower diffusional uptake. Increasing E3 pH to 8 increased the uncharged form, allowing greater uptake and thereby significantly lowering ketamine EC50 values. This has implications for other drugs used in anesthesia practice, such as opioids, and indicate that the pH of the embryo media must be tailored to the compound.

Following the creation of the model, the behavior patterns seen with both the tap and electrical stimuli demonstrated an ability for the 5 dpf zebrafish to display complex behaviors. For example, the we observed a freezing behavior seen in the larval fish reminiscent of that seen in mustard oil. Following addition of mustard oil, which is activates the TRPA1 chemosensor, larvae show a short burst of movement, followed by a reduction to minimal levels. Baseline movement is not restored until the chemical is removed. A similar biphasic behavior occurred after ELEC. Adult zebrafish also freeze on exposure to alarm pheromone and the scototaxis test (light/dark preference)^41^. Freezing may be a behavioral indicator of anxiety^41^, which is well developed at 5–7 dpf^42^. Whether freezing behavior after mustard oil or electrical stimulus reflects anxiety is not clear.

We do know, however, a great deal about the neural mechanisms of the TAP response and this can be compared to our observed ELEC responses. TAP is sensed through the zebrafish hair cells and transmitted to the hindbrain, specifically Mauthner cells^40^. Mauthner cells are bilateral large bundles of myelinated neurons that induce the fish to move to the contralateral side causing the fish swim way from the TAP. TAP does not involve higher cognitive functions, as it is a reflex. Interestingly, freezing behavior may also rely on Mauthner cells. ELEC signaling pathways are still unknown, but seem qualitatively similar to mustard oil.

When we placed the zebrafish into multiple anesthetics, we found that they responded at clinically relevant concentrations. To our knowledge, this is the first measurement of volatile anesthetic sensitivity in the larval zebrafish. The EC50 values are remarkably similar to those measured in mammalian species, including human (Table 1). Also similar is the large variation of movement responses at low isoflurane concentration, which appears to produce “excitation” in some animals, as it does in adult fish^43^ and in humans^44^.

We also found that zebrafish larvae responded to the three injectable anesthetics at clinically-relevant concentrations, with the TAP having the highest EC50 in two of the three. To test if our different paradigms could differentiate between sedation and general anesthesia, we included dexmedetomidine, a sedative drug that does not cause general anesthesia. This drug showed no loss of movement to TAP, but did show loss to SPONT and ELEC. Interestingly, in previous studies dexmedetomidine did show complete loss of movement while using the light reflex^23^. When comparing the values obtained from Yang et al.^23^ using 7 dpf zebrafish larvae, the spontaneous movement numbers were very similar and the response to the light stimulus gave an EC50 between that of SPONT and ELEC (Table 1). This would suggest that the light stimulus may model a lighter sedation than the ELEC or TAP but more than the SPONT. The exception to this point is etomidate, where our studies required significantly more drug to suppress spontaneous movement. Both sets of data are within the similar to what is seen in human studies^26–30^ (Table 1).

Taken together, our data indicate that the zebrafish is a robust and tractable vertebrate model system that responds to both inhaled and IV anesthetics at concentrations and phenotypes similar to humans and other mammals. The ability to reproduce states from sedation to anesthesia in such a tractable model will be useful when deploying genetic and imaging approaches to determine molecular and neuronal substrates underlying each state. Additionally, using this simple, quantitative, robust loss of movement paradigm will allow for easy reproducibility in other laboratories.

## Methods

### Animals

All zebrafish experiments were conducted in accordance to animal use protocols approved by the institutional animal care and use committee (IACUC). The adult zebrafish were maintained at the University of Pennsylvania’s aquatic facility and overseen by the University Laboratory Animal Resources (ULAR). They were maintained using standard husbandry conditions with 13–11 h light dark cycle and fed brine shrimp daily^45^. Tubingen long fin wild type animals were used for all experiments. The adults were mated and the embryos collected and maintained in the 13–11 h light dark cycle until they were 5 days post fertilization (dpf). Experiments were performed using multiple tanks of mating pairs to ensure adequate biological diversity.

### Behavioral experiments

**Figure 5:**
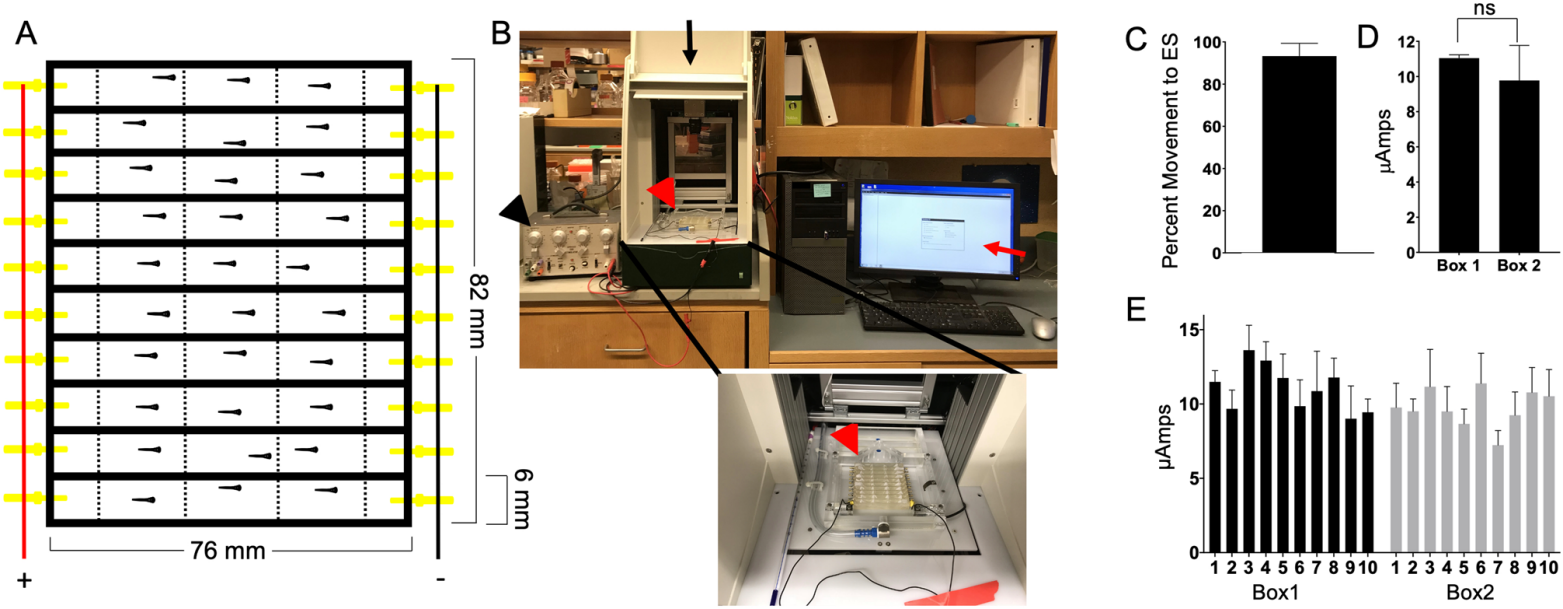
Electrical stimulation apparatus made to create a low amplitude, consistent noxious stimulus. (**A**) The box was 3D printed using free software (TinkerCad) with five chambers. The first and last chamber held a gold pin for the electrical current. The middle three chambers were for zebrafish larvae. The chambers were separated by a semi-permeable cloth membrane (dotted lines). This allowed liquid and, therefore, current, to flow but did not allow the larvae to move between chambers. (**B**) The apparatus (red arrowhead) attached to the Grass stimulator (black arrowhead) was placed within a Noldus DanioVision behavior box (black arrow) and a video of their movement was recorded (red arrow). (**C**) The zebrafish larvae moved 93.3% ± 6.1% of the time to the ELEC stimulus. n = 5 replicates with 12 zebrafish per replicate. (**D**, **E**) Two separate boxes were fabricated and there was no significant difference between the µAmps generated in the lanes of the two boxes. n = 3 replicates.

For all behavioral experiments, we used the DanioVision behavioral system (Noldus). Their software tracks and records the zebrafish larval movement within a chamber. Examples of raw data for all three behaviors are shown in S Fig. 3. The size of the chamber is defined prior to each experiment. All exposures were at room temperature (24–25 °C) in E3 media (5 mM NaCl, 0.17 mM KCl, 0.33 mM CaCl_2_, 0.33 mM MgSO4, pH 7.2). For each experiment, the zebrafish were equilibrated to the drug (minimum of 30 min), and to the environment of the chamber (minimum of 20 min). At the end of the experiment, the stimulus was administered, either single TAP (at a strength of six out of eight in the DanioVision system) or ELEC (11 µAmps for 200 ms). These stimuli elicited 90–100% movement of the 5 dpf no drug control larvae. The camera recorded motion at 15 frames per second, and the software converted the movement between frames as a distance moved. For the SPONT and TAP, the fish were in circular multi-well plates, either 24, 48 or 96, depending upon the drug administered. The type of plate used was particular to the drug being tested (see below for specific drugs). For the ELEC, all drugs were tested using the fabricated ELEC box (Fig. 5). All recordings were scanned to ensure the software identified the zebrafish larvae. There were instances when the larvae were not identified, and these fish were excluded from analysis. This was more common in the 96-well plates, per plate there was a loss of 0–5 larvae; whereas the 24 well plates had a loss of 1–2 larvae. These were primarily in the wells with higher concentrations of anesthetics, where the larvae did not move.

We used different lengths of time to record distance in each behavioral paradigm. To ensure a robust value for SPONT, we measured the distance moved over a period of 4 min, ending 1 min prior to the TAP. Movement in response to the TAP was measured over a period of 1 s. Finally, the ELEC response was measured over 2 s. Because this stimulus required us to manually trigger the stimulator, the increased time helped to adjust for human error. Control larval movement (no drug) was recorded for each experiment, and this distance was used to normalize all of the drug experiments.

### Fabricating the ELEC chamber

To use ELEC as a noxious stimulus, we created a chamber via three-dimensional (3D) printing using freely available software (tinkercad, https://www.tinkercad.com) (Fig. 5a). The box was 82 mm × 76 mm. Each lane is 6 mm wide and 10 mm deep. In total, there are ten lanes that accommodates 2 ml of fluid. Each of the ten lanes were isolated from the next. Each lane has a 3 mm gold pin on each end used to conduct the electricity. It was decided to use gold over other metals to prevent rusting and stainless steel did not conduct enough electricity within the small volume and relatively low ion environment of the chamber. The ten lanes are connected in series via a wire soldered onto the pin creating a positive and negative side (Fig. 5a).

To increase the number of larvae that could be tested at a given time, the lanes were divided into five separate wells. To allow electrical movement between wells but keep the zebrafish isolated within a single well, a semipermeable, fine weaved, white fabric (purchased from a local craft store) was used to create the five chambers. The fabric on the sides were curved to prevent the zebrafish larvae from hiding in a corner. The pin could not be placed in the same well as the zebrafish or the DanioVison software did not correctly identify the zebrafish larvae within the well. Therefore, the middle three wells contained a single zebrafish larva. This allowed for up to ten different concentrations of anesthetic to be tested with three larvae per concentration (Fig. 5a,b). The electrical stimulus was given using a grass stimulator, which was set at 20 V over 200 ms. A single stimulus achieved 93.3% ± 6.1% movement without visible signs of tissue damage on the larvae (Fig. 5c). Each well received approximately 11 µAmps of current and there was no significant difference between lanes or between the two boxes created (p > 0.05) (Fig. 5d,e).

### Anesthetic drug dilution and administration

All drug dilutions were made on the day of the experiment and any leftover drug solutions were discarded. They were diluted in standard E3 embryo water except for ketamine and the pH experiments. Those experiments were done with standard E3 plus 5 mM HEPES at pH 2–9. All larvae were left to equilibrate in the drug for a minimum of 30 min, the last 20 min of which were in the behavioral chamber. To accurately create the dilutions of the anesthetics used, specific protocols had to be followed depending upon the drug.

First, the inhaled anesthetics, halothane, isoflurane and sevoflurane, all used the following protocol. To ensure an accurate and reproducible concentration for the inhaled anesthetics, all equipment was glass and the dilution was drawn into Hamilton Gas-Tight syringes to avoid losses. We used halothane, isoflurane and sevoflurane (Sigma-Aldrich), which were stored at 4 °C. A stock dilution of 5 mM was made in glass scintillation vials, again using Hamilton syringes. Vials contained 22 ml of E3 embryo water, which left minimal air space so as not to lose drug. The solution was vortexed and sonicated repeatedly over 20 min. Each subsequent dilution was also made in the glass vials with a final volume of 22 ml. Once the 5 mM stock was opened, all subsequent dilutions were made at the same time using a Hamilton syringe to measure the volume and any remaining liquid was discarded. Each dilution was vortexed. For the behavioral analysis, the zebrafish were placed in a glass 96 well plate (JG Finneran Associates). All embryo water used to transfer the larvae was removed and Dow Corning high vacuum grease (Sigma-Aldrich) was placed around each well to ensure an airtight seal. Using a Hamilton syringe, each well was filled slightly over the top of the well with the anesthetic dilution. A glass cover slip was placed over the wells and a seal was made with the grease. The over-filled well ensured that there was no air between the well and the cover slip. The animal was equilibrated with the anesthetic for 30 min and then tested for response to stimuli.

Being highly hydrophobic drug, all propofol dilutions were made in glass scintillation vials to prevent adsorption of the propofol onto plastic. We used neat propofol (Sigma-Aldrich) which was initially diluted into 100% DMSO at 100 mM. This stock was subsequently diluted for each experiment, the maximal DMSO in an experimental dilution was 0.1%, which is 25-fold less than a toxic dose (above 2.5%) of DMSO^46^. For the experimental dilution, 20 µl of to DMSO stock was placed into 20 ml of E3. The dilution was vortexed and sonicated for 5–10 min, and the concentration confirmed by spectrophotometry, using an extinction coefficient of 1600/M at 270 nm^47^. When measuring movement for SPONT or after TAP, culture plates with round wells were used. However, the 48 and 96 well plastic plates held too small a volume to prevent significant adsorption of the propofol, so the 24 well plates were used with a minimum volume of 1.5 ml of propofol. A single larva was placed in each well and the E3 removed, prior to filling with propofol solution.

Etomidate is similarly hydrophobic, so the stock (2 mg/ml in 35% glycerol), is further diluted to 200 µM in 2% glycerol, which is safe for use with larval zebrafish^46^. No drug control also contained 2% glycerol. When testing the SPONT and TAP with etomidate, a 48 well plate was used, since etomidate does not adsorb to plastic to the same degree as propofol.

Ketamine and dexmedetomidine are more hydrophilic drugs and, therefore, were directly dissolved in E3. Both drugs were tested for SPONT and TAP using the 48 well plate. Ketamine was diluted into either E3 or E3 plus 5 mM HEPES at pH 8 for the behavioral experiments and the HPLC (see below).

Stock mustard oil (allyl isothiocyanate) was prepared at 5 mM in E3, and then subsequently diluted to the 200 µM concentration used experimentally. Since mustard oil needed to be acutely added to the wells, these experiments were not equilibrated in the behavior system prior to data collection. The zebrafish were placed within a 24 well plate with 1 ml of E3 and 40 µl of the 5 mM mustard oil was abruptly added to the well. Imaging system started as quickly as possible.

### High performance liquid chromatography (HPLC)

The HPLC system consisted of gold 126 solvent modules (Beckman Coulter), a UV spectrophotometer at 210 nm and an analytical C18 column (EclipseXDB-C18 250 mm × 4.6 mm > D., 5 µm particle size from Agilent Technologies). The mobile phase was a mixture of acetonitrile and 0.02 M phosphate buffer (pH 4) at 23:77 vol/vol and was eluted at 1.0 ml/min. Chromatic peaks for ketamine were identified by the retention time using the standard ketamine curves.

A standard ketamine curve was made prior each experiment. Known concentrations of ketamine at 2.5, 5, 10, 15 and 20 µM was diluted in methanol from a stock of 0.042 mM, and each was injected into the HPLC. The standard curve was constructed by plotting the height of the ketamine peak against the known concentrations and fit via a linear regression analysis.

To establish the equilibrium time for ketamine uptake into the zebrafish, an exposure time course of 0, 15, 30, 45 and 60 min to 200 µM ketamine was performed. We prepared the E3 at two pH values, 7.2 or 8.0, in 5 mM HEPEs buffer. To allow for accurate timing and decrease pipetting errors, we 3D printed baskets (tinkercad) with mesh bottoms that fit into 24 well plates. The wet baskets were each weighed and recorded. Then, 20–25 5 dpf larval zebrafish were placed in the baskets. The baskets were moved into a well with 200 µM ketamine made at the two pH values (spanning the ketamine pKa), with a single basket removed at each time point. The basket was washed quickly in E3 to remove any ketamine, blotted and then weighed to allow determination of zebrafish mass. The fish were then moved from the basket into an Eppendorf tube with 100 µl of deionized water, and stored at − 80 °C.

To prepare for the HPLC, 200 µl of acetonitrile was added to the 100 µl of deionized water plus zebrafish, homogenized and centrifuged at 4 °C at 20,000*g* for 20 min. Twenty µl of the supernatant was injected into the HPLC, and ketamine chromatographic peaks identified and measured. The standard curve allowed us to determine ketamine mass, which was then normalized to zebrafish tissue mass.

### Statistics

All experiments were replicated a minimum of three times. When using larval zebrafish, the minimum number of zebrafish per replicate was 8. To obtain the EC50 of each drug, the percent of control distance moved was plotted against the log of the drug concentration using GraphPad Prism software version 8.4.1 (https://www.graphpad.com/). We used nonlinear regression analysis to create a sigmoidal curve. The curves were constrained to require the top of the curve to be 100% movement and the bottom to be 0% movement. However, for the isoflurane, the lowest concentration, 10 mM, was excluded from the analysis due to its large variance and height above the 100% mark due to behavioral excitation. All graphs shown use standard deviation, with the 95% confidence intervals specified in the tables. For data in Figs. 2, 3 and 5, where p values were required, they were obtained using the Wilcoxon matched pairs signed rank test for nonparametric pairwise comparison.

## Supplementary information

Supplementary Information.
