# Supplementary material for: A vertebrate model to reveal neural substrates underlying the transitions between conscious and unconscious states
Source: Sci Rep. 2020 Sep 25;10:15789. doi: 10.1038/s41598-020-72669-1 (PMC7519646; doi:10.1038/s41598-020-72669-1)
Supplement: Supplementary file 1 — Supplementary Information. [file 41598_2020_72669_MOESM1_ESM.docx]

A vertebrate model to reveal neural substrates underlying the transitions between conscious and unconscious states.

Victoria M Bedell^1*^, Qing C. Meng^1^, Michael A. Pack^2^, Roderic G. Eckenhoff^1^

1. Department of Anesthesiology and Critical Care, University of Pennsylvania, Philadelphia, United States.
2. Department of Medicine, University of Pennsylvania, Philadelphia, United States.

[*Victoria.bedell@pennmedicine.upenn.edu](mailto:*Victoria.bedell@pennmedicine.upenn.edu)

**Supplemental Table 1:**

|  | **pH 8** | | **pH 7.2** | |
| --- | --- | --- | --- | --- |
| **Behavior** | **EC50 (µM)** | **95% CI (µM)** | **EC50 (µM)** | **95% CI (µM)** |
| SPONT | 7.7 | 3.8-16.4 | 9.2 | 4.2-18.6 |
| TAP | 152.0 | 86.4-272.5 | 696.1 | 360-1348 |
| ELECT | 36.3 | 19.7-66.8 | 681.2 | 462-988 |

**Supplemental Table 1: Comparing the EC50 of ketamine at pH 8 versus pH 7.2.** For all three behaviors, more ketamine is required to cause loss of movement in the pH 7.2 versus the pH 8. However, this is seen most dramatically in the ELEC, which required a 20-fold higher concentration to reach the EC50.


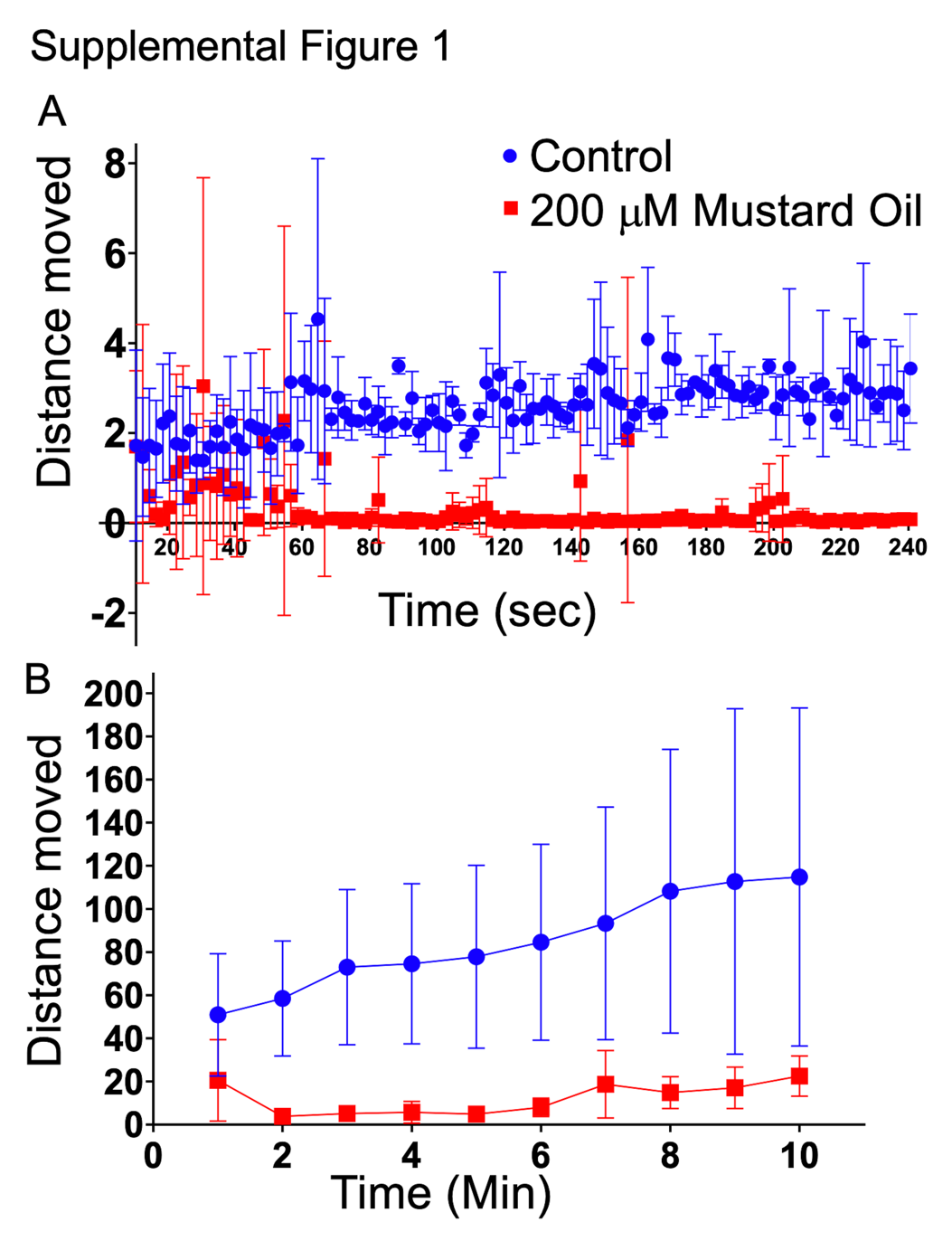


**Supplemental Figure 1: Movement data of a known noxious stimulus, mustard oil.** A) Total distance moved over 2 seconds directly following addition of the mustard oil. Both the control and mustard oil treatments show very high error bars for the first minute. B) Total distance moved over 1 minute for the first 10 minutes. The mustard oil stays close to minimal movement. However, the control continued to increase in movement and have very large error bars for the first few minutes within the behavior chamber. All graphs were created with 5 replicates per point and 8-12 zebrafish larvae used per replicate.


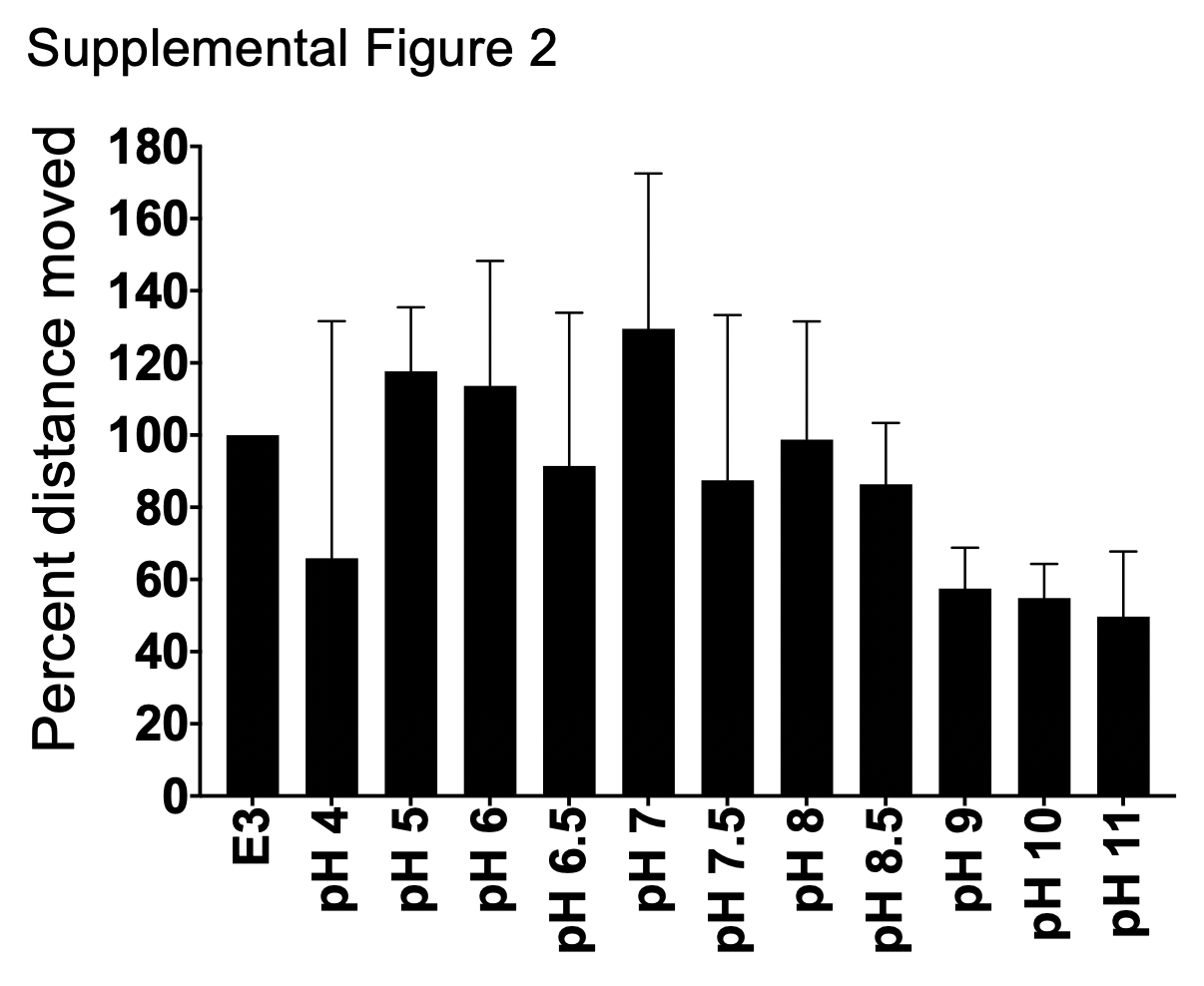


**Supplemental Figure 2: Effect of pH on baseline movement.** Between the pH of 5-8.5, no significant changes in movement were seen either due to pH or due to the 5 mM Hepes added. At the extreme pHs, 4, 9, 10 and 11, there is a decrease in the baseline movement. At pH 2 and 3 all larvae were dead (data note shown). All graphs were created with 3 replicates per point and 8-12 zebrafish larvae used per replicate.


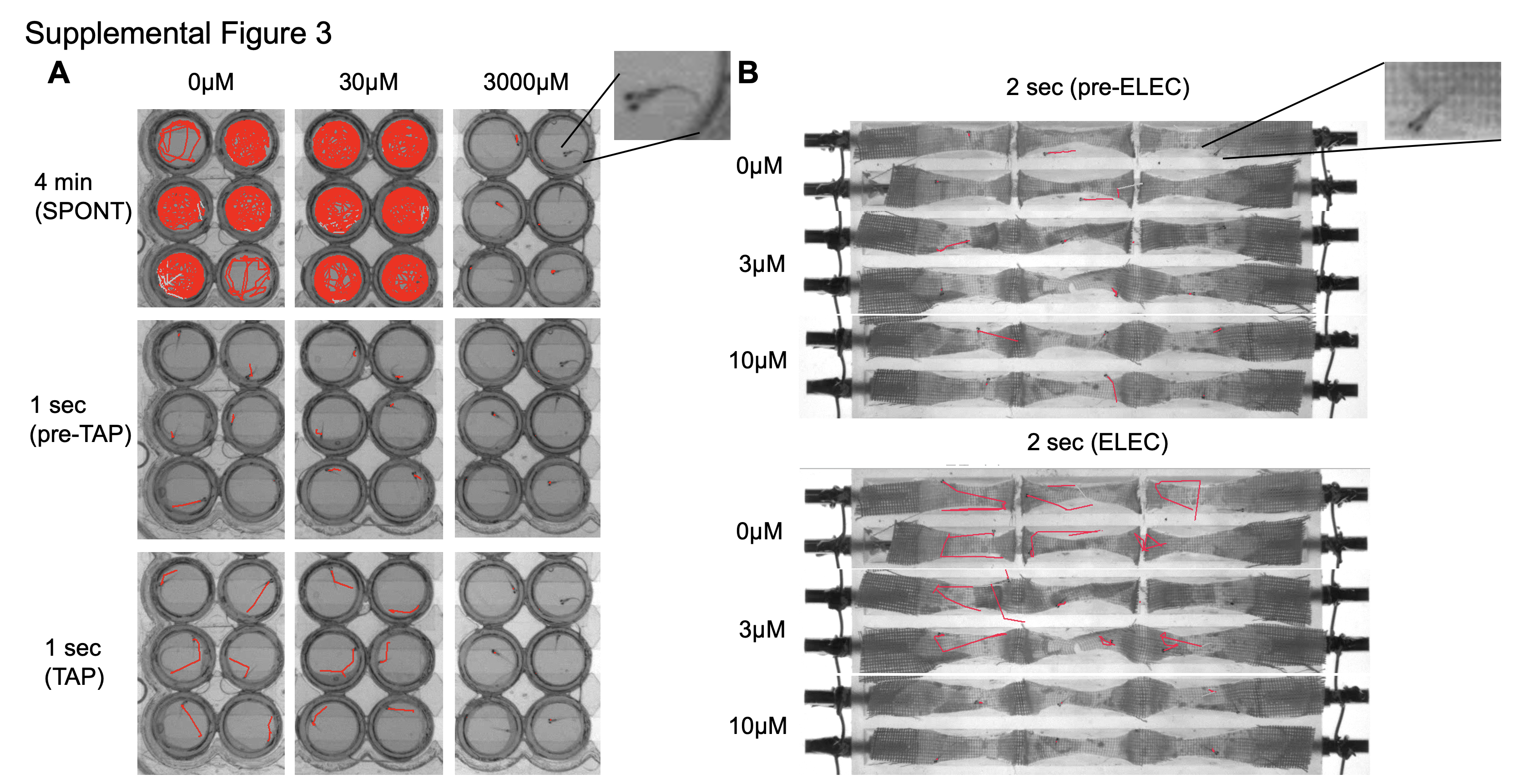


**Supplemental Figure 3: Sample behavior data.** A) An example of how SPONT and TAP stimuli appear in a 96-well plate, specifically with halothane. The expanded image shows the zebrafish within the well. The SPONT movement is over 4 minutes with no drug and low dose drug showing movement, seen by the red within the wells, and 3000μM halothane showing minimal movement. The movement in 1 second following the TAP is much higher than the baseline movement (pre-TAP). Additionally, all movement to the TAP is lost at 3000μM halothane. B) An example of ELEC with increasing doses of propofol. The expanded box shows the zebrafish within the ELEC chamber. The baseline movement is lower than the post stimuli movement with decreasing movement seen as the amount of propofol increases.
